# A Gunshot Wound of the Heart

**Published:** 1903-01-10

**Authors:** 


### A GUNSHOT WOUND OF THE HEART.

ANOTHER case of wound of the heart has recently been reported by Dr. Hammond [1] which, although a fatal result ensued, gave evidence of the power possessed by the heart of doing its work even when seriously injured. The patient was a man 51 years of age who had been accidentally shot in the chest, the wound of entrance being one and a half inches above the ensiform cartilage and about the same distance to the left of the sternum. The patient did well for nearly three weeks, when he was suddenly attacked with vomiting and died almost immediately. On post-mortem examination it was found that the bullet had passed through the pericardium and through the apex of the heart, making a wound about a quarter of an inch in depth, after which it had passed through the pleura and out of the chest behind, being found in the subcutaneous tissues of the back. The entire heart was enveloped in a well-organised blood-clot, and about a gallon of serum and blood was removed from the pericardium and pleural cavities. Possibly the presence of this large amount of fluid in the chest had much to do with the sudden death. It is an interesting question how far the blood-clot would have been ultimately absorbed had the continuance of life been rendered possible by the removal of the fluid. So far as the wound went, the heart's action does not appear to have been seriously impaired until the final strain took place.

[1] Annals of Surgery, Oct., 1902.

